# Isolation of *Aspergillus *spp. from the respiratory tract in critically ill patients: risk factors, clinical presentation and outcome

**DOI:** 10.1186/cc3488

**Published:** 2005-03-11

**Authors:** José Garnacho-Montero, Rosario Amaya-Villar, Carlos Ortiz-Leyba, Cristóbal León, Francisco Álvarez-Lerma, Juan Nolla-Salas, José R Iruretagoyena, Fernando Barcenilla

**Affiliations:** 1 Department of Intensive Care Medicine, Hospital Universitario Virgen del Rocío, Sevilla, Spain; 2 Department of Intensive Care Medicine, Hospital Universitario Virgen del Rocío, Sevilla, Spain; 3 Department of Intensive Care Medicine, Hospital Universitario Virgen del Rocío, Sevilla, Spain; 4 Department of Intensive Care Medicine, Hospital Universitario de Valme, Sevilla, Spain; 5 Department of Intensive Care Medicine, Hospital Universitari del Mar, Barcelona, Spain; 6 Department of Intensive Care Medicine, Hospital Universitari del Mar, Barcelona, Spain; 7 Department of Intensive Care Medicine, Hospital de Cruces, Bilbao, Bikzakia, Spain; 8 Department of Intensive Care Medicine, Hopsital Universitari Arnau de Vilanova, Lleida, Spain

## Abstract

**Introduction:**

Our aims were to assess risk factors, clinical features, management and outcomes in critically ill patients in whom *Aspergillus *spp. were isolated from respiratory secretions, using a database from a study designed to assess fungal infections.

**Methods:**

A multicentre prospective study was conducted over a 9-month period in 73 intensive care units (ICUs) and included patients with an ICU stay longer than 7 days. Tracheal aspirate and urine samples, and oropharyngeal and gastric swabs were collected and cultured each week. On admission to the ICU and at the initiation of antifungal therapy, the severity of illness was evaluated using the Acute Physiology and Chronic Health Evaluation II score. Retrospectively, isolation of *Aspergillus *spp. was considered to reflect colonization if the patient did not fulfil criteria for pneumonia, and infection if the patient met criteria for pulmonary infection and if the clinician in charge considered the isolation to be clinically valuable. Risk factors, antifungal use and duration of therapy were noted.

**Results:**

Out of a total of 1756 patients, *Aspergillus *spp. were recovered in 36. Treatment with steroids (odds ratio = 4.5) and chronic obstructive pulmonary disease (odds ratio = 2.9) were significantly associated with *Aspergillus *spp. isolation in multivariate analysis. In 14 patients isolation of *Aspergillus *spp. was interpreted as colonization, in 20 it was interpreted as invasive aspergillosis, and two cases were not classified. The mortality rates were 50% in the colonization group and 80% in the invasive infection group. Autopsy was performed in five patients with clinically suspected infection and confirmed the diagnosis in all of these cases.

**Conclusion:**

In critically ill patients, treatment should be considered if features of pulmonary infection are present and *Aspergillus *spp. are isolated from respiratory secretions.

## Introduction

*Aspergillus *is a genus of mitosporic fungi, some species of which are known to cause infections in humans, particularly *Aspergillus fumigatus *(85% of cases) followed by *A flavus *and *A niger *[1]. *Aspergillus *spp. are responsible for a broad spectrum of illnesses, from saprophytic colonization of the bronchial tree to rapidly invasive and disseminated diseases. Invasive aspergillosis remains a major cause of morbidity and mortality in immunosuppressed patients with profound granulocytopenia secondary to haematological malignancies, or solid organ and bone marrow transplantation. Outbreaks of aspergillosis in patients admitted to intensive care units (ICUs) have been reported [2]. *Aspergillus *spp. can also cause pneumonia in ICU patients without classical predisposing factors, as well as community-acquired pneumonia in otherwise immunocompetent healthy individuals [3,4].

Because the mortality rate with invasive aspergillosis remains high, even in the face of therapy, the work up must be prompt and aggressive. The diagnosis of invasive pulmonary aspergillosis is difficult because definitive diagnosis is based on histological documentation of typical hyphae and a culture positive for an *Aspergillus *sp. Uncertainty in disease definition is a key contributor to the controversy regarding the optimal method for establishing the diagnosis of invasive infection.

Standard definitions of opportunistic fungal infections in immunocompromised patients with cancer and haematopoietic stem cell transplants were recently proposed [5]. 'Proven' aspergillosis requires histopathological or cytopathological examination showing hyphae with evidence of associated tissue damage, or a positive culture result from a sample obtained using sterile technique along with suggestive clinical or radiological evidence of infection. In addition, 'probable' aspergillosis requires the presence of risk factors in the host, isolation of an *Aspergillus *sp. and suggestive clinical or radiological findings; 'possible' aspergillosis requires the presence of risk factors in the host and isolation of an *Aspergillus *sp., or suggestive clinical and radiological findings [5]. Serology is not useful in the diagnosis of aspergillosis, and data regarding the clinical utility of detection of *Aspergillus *antigenaemia is limited to patients with neutropenia [6].

Treatment is mandatory in severely immunocompromised patients (those with neutropenia or prolonged use of immunosuppressants) with suggestive clinical manifestations or isolation of *Aspergillus *spp. in respiratory secretions. However, the therapeutic approach is not well defined in critically ill patients without neutropenia or transplantation in whom *Aspergillus *spp. are cultured in bronchial secretions [7]. Therefore, using data from a large multicentre study designed to assess risk factors and the impact of isolation of fungi in ICU patients, the present study was performed with the following objectives: to determine risk factors for respiratory isolation of *Aspergillus *spp.; to assess clinical features, treatment and outcomes in patients with *Aspergillus *spp. recovered from respiratory secretions; and to evaluate the correlation between isolation of *Aspergillus *spp. in respiratory samples and histopathological findings.

## Materials and methods

### Study population

A total of 1765 patients older than 18 years of age who were admitted for at last 7 days to 73 medical/surgical ICUs in certain Spanish hospitals between May 1998 and January 1999 were included in the study. The institutional review board of each hospital approved the protocol and waived the need for informed consent.

### Design

This was a prospective, cohort, observational, multicentre study. Based on diagnosis at the time of ICU admission, patients were classified as medical, surgical, or trauma. The severity of illness on ICU admission was calculated using the Acute Physiology and Chronic Health Evaluation (APACHE) II scoring system [8]. The definitions of severe sepsis and septic shock used were those of the American College of Chest Physicians/Society of Critical Care Medicine Consensus Conference [9].

In all patients, samples obtained from sputum, tracheal aspirates (intubated patients), urine, pharyngeal exudates and gastric aspirates were cultured for fungi each week. The initial samples were obtained 8 days after admission to the ICU and once a week thereafter. Other samples of peripheral blood or from other infectious foci were obtained at the discretion of the attending physician. Samples were processed by the various reference clinical microbiology laboratories of the participating hospitals using standard procedures, including Sabouraud agar culture and BACTEC method (Becton Dickinson Diagnostic Instrument Systems, Paramus, NJ, USA), for the isolation of fungal species. The A20C system (Byomerieux, Lyon, France) was used for species identification. *Candida *infection was defined as recovery of *Candida *spp. from blood samples (in one or more culture bottles), or evidence of endophthalmitis, or a positive culture of tissue specimens or peritoneal fluid culture, or obstruction of the urinary tract by fungal balls.

### Risk factors

Various risk factors before ICU admission and during the ICU stay were recorded. These are summarized in Table 1.

### Clinical features

Patients with *Aspergillus *spp. isolated from respiratory samples were retrospectively evaluated. The clinical significance of recovery of *Aspergillus *spp. was determined individually by the physician in charge, who established whether isolation of *Aspergillus *spp. represented a case of colonization or infection. Colonization was deemed to be present when the patient did not fulfil criteria for pneumonia; if the patient fulfilled criteria for pneumonia and the clinician in charge considered the isolation of *Aspergillus *spp. to be clinically valuable, then the patient was considered to be infected. Specific recommendations regarding therapeutic approach when fungi were isolated from culture were not given, and so the decision regarding antifungal treatment was made on an individual basis by the physician in charge. In patients treated with antifungal drugs, adverse events, clinical cure and microbiological eradication (weekly cultures becoming negative) were recorded. For each patient in whom an *Aspergillus *sp. was detected, clinical data as well as radiographic and computed tomography findings were retrospectively recorded by means of a questionnaire completed by the clinician in charge. Radiographic findings included normal chest radiograph, lobar consolidation, unilateral consolidation, bilateral consolidation and ill-defined nodules [10].

Patients were followed until discharge from the hospital or death during the hospital stay. In patients who died with proven fungal infection or with high suspicion of fungal infection, an autopsy examination was sought.

### Statistical analysis

Qualitative variables are expressed as the percentage distribution in each category, and quantitative variables are expressed as mean ± standard deviation in normally distributed variables or median (range) when the distribution was not normal. The Student's t-test or the Mann–Whitney U-test was used for the comparison of categorical and normally distributed and non-normally distributed variables, respectively. Analysis of variance or the Kruskal–Wallis test was used in the comparison of three groups. The χ^2 ^test or the Fisher's exact test was used in the comparison of categorical variables. A comparison of risk factors for the isolation of *Aspergillus *spp. between groups of patients with *Aspergillus *spp., patients with *Candida *spp. infection, and noncolonized, uninfected patients was conducted. For this purpose, a binary logistic regression analysis prior to the bivariate analyses was performed. Variables were included in the model if *P *≤ 0.05. Results are expressed as odds ratio (OR), 95% confidence interval (CI). *P *< 0.05 was considered statistically significant. Statistical analysis was performed using the Statistical Package for the Social Sciences (SPSS) for Windows (version 11.5; SPSS Inc., Chicago, IL, USA).

## Results

The study population included 1765 patients (1178 [67%] men; mean [± standard deviation] age 57.8 ± 17.3 years). Underlying diseases were classified as medical in 44% of patients, surgical in 47% and trauma in 9%. A total of 1045 patients were classified as colonized or infected with fungi, and 720 were classified as noncolonized, uninfected patients. Colonization with *Candida *spp. was diagnosed in 880 (49.8%) patients, *Candida *spp. infection in 105 (5.9%), and infection with fungi other than *Candida *spp. in 60 (3.4%). In this group of 60 patients, in whom fungi other than *Candida *spp. were isolated, *Aspergillus *spp. were recovered in 38 (63.3%). An *Aspergillus *sp. was isolated from respiratory secretions in 36 patients (tracheal aspirate 35, sputum 1). *A fumigatus *was isolated in 35 patients and *A niger *in one. The length of ICU stay was similar between patients infected with *Aspergillus *spp. and those infected with *Candida *spp. (32.1 ± 21.4 days versus 32.8 ± 22.6 days), but it was significantly longer than in noncolonized, uninfected patients (18.4 ± 14.1 days; *P *< 0.001; Table 2).

Compared with noncolonized, uninfected patients, patients with *Aspergillus *spp. infection had significantly greater in-hospital mortality (69.4% versus 33%; *P *< 0.001) and ICU mortality (52.8% versus 24.7%; *P *< 0.001) rates. Patients with *Candida *spp. infection also had significantly greater in-hospital mortality (60.9% versus 33%; *P *< 0.001) and ICU mortality (53.3% versus 24.7%; *P *< 0.001) rates than did noncolonized, uninfected patients.

### Risk factors

The frequency of risk factors for fungal infection before ICU admission were similar in the three groups of patients (Table 2), except for significantly higher rates of chronic obstructive pulmonary disease (COPD), immunosuppression and transplantation in the patients with *Aspergillus *infection, and a greater prevalence of solid neoplasms in the patients with *Candida *infection. With regard to risk factors present during the ICU stay, neutropenia and treatment with steroids were significantly more frequent in the *Aspergillus *group, and total parenteral nutrition was significantly more common in the *Candida *group (Table 2). Duration of steroid administration was also significantly longer in the *Aspergillus *group (Table 3). In multivariate analysis, independent factors significantly associated with recovery of *Aspergillus *spp. compared with noncolonized, uninfected patients were treatment with steroids (OR = 4.5, 95% CI = 1.73–11; *P *= 0.002) and COPD (OR = 2.9, 95% CI = 1.06–8.08; *P *= 0.03). When comparisons with patients with *Candida *infection were performed, immunosuppression (OR = 12.9, 95% CI = 1.34–25; *P *= 0.001), neutropenia (OR = 9.4, 95% CI = 1.9–19.9; *P *= 0.02) and COPD (OR = 9.2, 95% CI 1.36–62.5; *P *= 0.02) emerged as independent factors significantly associated with isolation of *Aspergillus *spp.

### Clinical characteristics

*Aspergillus *spp. were isolated from respiratory samples in severely ill patients, with a mean APACHE II score on ICU admission of 21.6 ± 6.9 and a mean age of 58.7 ± 16.6 years. Apart for eight patients with *Aspergillus *infection, the remaining 28 patients had debilitating underlying disorders, with COPD (*n *= 16), immunosuppression (*n *= 20) and chronic renal failure (*n *= 10) being the most common. During their stay in the ICU, 25 patients received steroids and all but one were mechanically ventilated. The mean length of ICU stay before isolation of *Aspergillus *spp. was 32.1 ± 21.4 days. Previous use of fluconazole was recorded in eight of the 36 patients (22.2%) with isolation of *Aspergillus *spp., and in 41 of the 105 patients (39%) with invasive candidiasis.

In 14 patients without clinical symptoms of pneumonia, isolation of *Aspergillus *spp. was interpreted by the clinician in charge as colonization. In two patients *Aspergillus *spp. were recovered 24 hours before the patient's death, and so the clinical manifestations could not be evaluated. The remaining 20 patients had signs of severe sepsis or septic shock unresponsive to broad-spectrum antibiotics in association with clinical manifestations suggestive of pneumonia. In these cases, isolation of *Aspergillus *spp. was interpreted to represent infection, and treatment with antifungal agents was started. In seven of these patients, however, bacteria in association with *Aspergillus *spp. were isolated from the tracheal aspirates, including *Pseudomonas aeruginosa *(*n *= 2), *Klebsiella pneumoniae *(*n *= 1), *Acitenobacter baumannii *(*n *= 1), *Stenotrophomonas maltophilia *(*n *= 1), coagulase-negative *Staphylococcus *spp. and *Haemophilus *spp. (*n *= 1). The most frequent radiographic findings were unilateral consolidation and bilateral consolidation.

### Treatment and outcome

In the group of 14 patients with *Aspergillus *colonization, the in-hospital mortality rate was 50% (three patients died in the ICU). Eleven patients were not treated with antifungal drugs, although risk factors were present in seven. Liposomal amphotericin B was prescribed to three patients (one of these patients with predisposing risk factors died in the ICU). The mean cumulative dose of amphotericin B lipid formulation was 3100 mg and the mean duration of treatment was 9 days.

Of the 20 patients with *Aspergillus *spp. infection 16 died, yielding an in-hospital mortality rate of 80%. All patients were given amphotericin B except one patient, who was treated with intraconazole. Details of treatment are shown in Table 4. The mean APACHE II score at the beginning of antifungal treatment was 22.7 ± 8, as compared with 14.3 ± 2.3 in treated patients colonized with *Aspergillus *spp. The first choice antifungals were amphotericin B deoxycholate (administered to eight patients), liposomal amphotericin B (eight patients) and amphotericin B lipid complex (three patients). Two patients treated with amphotericin B deoxycholate developed renal failure and treatment was changed to liposomal amphotericin B in one and amphotericin B lipid complex in the other. One patient initially treated with amphotericin B lipid complex was switched to liposomal amphotericin B because of persistence of infection, with positive cultures, after 2 weeks of treatment. After 3 weeks of treatment with liposomal amphotericin B, cultures were negative. Eleven patients died, and in the remaining nine patients treatment was discontinued after clinical cure. Mean duration of treatment in these nine patients was 18 days (range 8–35 days). Clinical resolution of symptoms was achieved with amphotericin B deoxycholate only in one patient and with the lipid formulation in eight (*P *< 0.05).

Autopsy was performed in five patients with *Aspergillus *spp. infection. In all cases the examination revealed characteristic hyphae elements within the lung parenchyma with vascular invasion, which is compatible with the diagnosis of invasive aspergillosis. All were COPD patients and had been treated with corticosteroids in the ICU. One patient had a lung cancer. None of these five patients had neutropenia or haematological malignancy.

## Discussion

This is the largest study to date in which *Aspergillus *spp. were isolated from respiratory secretions in a cohort of critically ill patients, including a large number of immunocompetent patients. In this group, isolation of *Aspergillus *spp. mostly occurred in those with COPD who were treated with steroids during their ICU stay. However, only 13.8% of patients had neutropenia – a classic risk factor for *Aspergillus *infection.

Various small series and case reports have shown that invasive aspergillosis commonly occurs in critically ill patients admitted to the ICU because of acute exacerbation of COPD and treated with intravenous corticosteroids [11-14]. In those patients steroids were given for a short period (1 week), whereas in our patients treatment was prolonged (3 weeks). In contrast, in a recent study of 250 patients with COPD admitted to the ICU because of acute respiratory failure [15], which did not report on the use of corticosteroids, *Aspergillus *spp. were not isolated in any respiratory sample. On the other hand, prior treatment with fluconazole was not associated with a higher rate of isolation of *Aspergillus *spp., as was previously reported in patients with neutropenia [16].

In one-third of cases in the present study recovery of *Aspergillus *spp. in respiratory secretions, in the absence of signs of pneumonia, was considered to represent colonization. However, three of these patients were given antifungal treatment because of underlying risk factors. An important finding of the study is that systemic antifungal agents were employed in patients with *Aspergillus *spp. colonization with clinical signs of respiratory infection, despite the fact that associated bacterial pathogens were cultured in almost one-third of cases. Although autopsies were performed in only five patients with *Aspergillus *infection, histopathological findings confirmed the clinical diagnosis in each case. Our findings are in agreement with those of a recent autopsy study [17] that confirmed the diagnostic value of *Aspergillus *spp. in respiratory secretions of COPD patients admitted to the ICU and treated with corticosteroids. In contrast, in a study conducted Petri and coworkers [18] in 435 non-neutropenic ICU patients, fungal colonization with *Aspergillus *spp. was found in 4% of cases, but in none of the patients was a diagnosis of invasive aspergillosis made.

In one study [19], because of the lack of reliable diagnostic tools, up to 60% of patients with invasive aspergillosis diagnosed at autopsy had not received antifungal treatment. Isolation of *Aspergillus *spp. from respiratory secretions has been regarded as being of limited usefulness in the antemortem diagnosis of invasive aspergillosis. In a study conducted in the 1980 s, Yu and coworkers [20] evaluated 108 patients in whom *Aspergillus *spp. were isolated from respiratory secretions, but invasive aspergillosis was not demonstrated in non-immunosuppressed patients. In a recent study [21], however, it was shown that malnutrition, diabetes mellitus, pulmonary disorder, or corticosteroid use were underlying risk factors for invasive aspergillosis in patients in whom *Aspergillus *spp. were isolated from respiratory secretions. On the other hand, invasive aspergillosis does not only occur in immunocompromised patients [3]. In a cohort of 439 non-ICU patients with invasive aspergillosis [22], nine had no apparent underlying conditions before diagnosis. Likewise, acute community-acquired pneumonia due to *Aspergillus *spp. – a rare infection – has been reported in 12 immunocompetent hosts [4].

It is well known that neutropenia is the main risk factor for aspergillosis because polymorphonuclear neutrophils and macrophages are the first immunological line of defence against *Aspergillus *spp. [6]. However, T-cell mediated, acquired immunity also plays a role in protecting against fungal infection [23]. Critically ill patients with prolonged stays in the ICU exhibit a complex decrease in immune function, with deactivation of macrophages and altered cellular response [24]. In addition, the immune function of peripheral neutrophils is influenced by acute hyperglycaemia [25]. Furthermore, it has been shown that corticosteroids suppress neutrophil action against *Aspergillus *hyphae [26]. These mechanisms may explain why *Aspergillus *infection occurs in ICU patients with a compensatory anti-inflammatory response syndrome or immunoparalysis during multiorgan failure but without any predisposing factors [27,28]; they may also account for the association between corticosteroid use and this invasive fungal infection.

Invasive aspergillosis in ICU patients carries a very high mortality [4,28,29], with an attributable mortality of 18.9% after adjusting for confounding factors [30]. In non-immunocompromised patients, the success of antifungal treatment depends on early diagnosis. However, because delayed diagnosis is the rule, if therapy is not promptly initiated then patients may die from the disease. Amphotericin B deoxycholate was the only therapeutic option in the past and was the antifungal agent used in series with a reported mortality of as high as 100%. In the present study, although there were no differences in in-hospital mortality according to antifungal drug used, clinical cure rates were higher in patients treated with amphotericin B lipid formulations. In two patients amphotericin B deoxycholate was withdrawn because of nephrotoxicity, which increases mortality significantly [31]. Although greater efficacy of amphotericin B lipid formulations compared with amphotericin B deoxycholate in the treatment of invasive aspergillosis has not been demonstrated [7], the use of the lipid formulations appears preferable, especially in critically ill patients, because of better tolerance [32]. New antifungal agents with good activity against *Aspergillus *spp. have recently become available. Initial treatment of invasive aspergillosis with voriconazole led to better response and improved survival than with the standard approach of initial therapy with amphotericin B [33]. Caspofungine was also effective as salvage therapy in invasive pulmonary aspergillosis, as compared with standard therapy [34].

One of the main limitations of the present study was the retrospective design, in which diagnostic and treatment approaches were not standardized. Also, there were few cases in which the clinical diagnosis of invasive pulmonary aspergillosis was confirmed by histopathological evaluation. Third, mortality rates may be biased by differences in antifungal treatments used at each centre. Nevertheless, the present data add valuable information regarding the significance of isolation of *Aspergillus *spp. from respiratory samples in critically ill patients.

## Conclusion

In summary, COPD and treatment with corticosteroids are major predisposing factors for *Aspergillus *spp. colonization/infection in critically ill patients. For this reason, in ICU patients with these risk factors, antifungal treatment should be considered in the presence of clinical features of pneumonia and isolation of *Aspergillus *spp. from respiratory secretions. In contrast, antifungal treatment should not be initiated when *Aspergillus *spp. are recovered from bronchial aspirates of critically ill patients without predisposing risk factors and in the absence of clinical and radiological signs of pneumonia. In these cases, isolation of *Aspergillus *spp. should be interpreted as colonization.

## Key messages

• 	COPD and treatment with corticosteroids, and neutropenia are major predisposing factors for respiratory colonization/infection with *Aspergillus *spp. in critically ill patients.

• 	In ICU patients with these risk factors, antifungal treatment should be considered in the presence of clinical features of pneumonia and isolation of *Aspergillus *spp. from respiratory secretions.

• 	The crude mortality associated with this entity is still very high.

## Abbreviations

APACHE = Acute Physiology and Chronic Health Evaluation; CI = confidence interval; COPD = chronic obstructive pulmonary disease; ICU = intensive care unit; OR = odds ratio.

## Competing interests

The author(s) declare that they have no competing interests.

## Authors' contributions

All of the authors were involved in designing the study and collecting data. JGM and RAV were involved in the statistical analysis. CL obtained funding. JGM drafted the manuscript, which was revised and approved by all of the authors.

## Figures and Tables

**Table 1:** Risk factors recorded before ICU admission and during ICU stay

Risk factor	Comments (where applicable)
Before ICU admission	

Surgery before ICU admission	Divided into urgent or elective
Diabetes mellitus	Only insulin-treated patients
COPD	Defined as the presence of a productive cough or expectoration for more than 90 days per year (but on separate days) and for more than 2 consecutive years, provided that a specific disorder responsible for these symptoms was not present
Chronic liver disease	With confirmation of the diagnosis by liver biopsy or in patients with signs of portal hypertension, such as oesophageal varices or ascites
Renal failure	Defined as need for haemodyalisis or peritoneal dialysis at the time of admission to the hospital
Severe heart failure	Defined as New York Heart Association functional class III or IV heart failure
Malignancy	Histological evidence required for a diagnosis of solid tumour and definitive diagnosis for the diagnosis of haematological malignancy
HIV infection	Defined as HIV-positive status
Neutropenia	Total leucocyte count ≤ 500/mm^3^
Immunosuppression	Altered immune status according to APACHE II criteria [8] or in case of a previous diagnosis (congenital or acquired)
Transplant recipients	Those patients receiving solid organ or bone marrow transplant
Chemotherapy	Use of cytotoxic agents for the treatment of a neoplasm or an autoimmune disease within 30 days before ICU admission
Radiotherapy	Radiation therapy within 30 days before ICU admission

During ICU stay	

Presence and duration of catheters	Urinary bladder, venous, or arterial catheter
Nutrition	Enteral or parenteral nutrition
Mechanical ventilation	
Dialysis	
Use of steroids	Patients treated with a daily dose equivalent to 20 mg prednisone
Neutropenia	Total leucocyte count ≤ 500/mm^3^
Drug use	Antimicrobial and antifungal agents

APACHE, Acute Physiology and Chronic Health Evaluation; COPD, chronic obstructive pulmonary disease; ICU, intensive care unit.

**Table 2:** Demographic data and risk factors for fungal infection in critically ill patients admitted to the ICU for more than 7 days

Variable	Isolation of *Aspergillus *spp.	*Candida *spp. infection	Noncolonized, uninfected patients	*P*
Patients (*n*)	36	105	720	
Age (years; mean ± SD)	58.7 ± 16.6)	59.5 ± 16.3	56.4 ± 17.4	NS
Men (*n *[%])	27 (75)	76 (72.4)	491 (68.2)	NS
APACHE II score (mean ± SD)	21.6 ± 6.9	18.5 ± 6.5	18.9 ± 8.1	0.05
ICU stay (days; mean ± SD)	32.1 ± 21.4)	32.8 (22.6)	18.4 (14.1)	<0.001
Risk factors before ICU admission (*n *[%])				
Diabetes mellitus	5 (13.9)	18 (17.1)	113 (15.7)	NS
COPD	16 (44.4)	16 (15.2)	179 (24.9)	0.002
Solid neoplasm	3 (8.3)	21 (20)	65 (9)	0.002
Hematological neoplasm	1 (2.8)	3 (2.9)	18 (2.5)	NS
Transplant recipient	3 (8.3)	0	3 (0.4)	<0.001
Immunosuppression	10 (27.8)	8 (7.6)	48 (6.7)	<0.001
Chronic renal failure	3 (8.3)	4 (3.8)	26 (3.6)	NS
HIV infection	1 (2.8)	2 (1.9)	9 (1.3)	NS
Chronic liver disease	2 (5.6)	3 (2.9)	29 (4)	NS
Severe heart failure	2 (5.6)	4 (3.8)	41 (5.7)	NS
Radiation therapy	0	4 (3.8)	7 (1)	NS
Chemotherapy	2 (5.6)	3 (2.9)	13 (1.8)	NS
Risk factors during ICU stay				
Arterial catheter	31 (86.1)	74 (70.4)	498 (69.1)	NS
Venous catheter	36 (100)	104 (99.0)	711 (98.6)	NS
Urinary catheter	36 (100)	100 (95.2)	703 (97.5)	NS
Mechanical ventilation	35 (97.2)	99 (94.2)	640 (88.8)	0.019
Total parenteral nutrition	20 (55.5)	90 (85.7)	274 (38.0)	<0.001
Haemodialysis	11 (30.6)	34 (32.3)	56 (7.8)	<0.001
Neutropenia	5 (13.8)	5 (4.7)	20 (2.8)	0.001
Steroids	25 (69.4)	25 (23.8)	139 (19.3)	<0.001
Antibiotic treatment	36 (100)	105 (100)	674 (93.5)	<0.001

APACHE, Acute Physiology and Chronic Health Evaluation; COPD, chronic obstructive pulmonary disease; ICU, intensive care unit; NS, not significant; SD, standard deviation.

**Table 3:** Duration (days) of intra-ICU risk factors for fungal infection

Variable	Isolation of *Aspergillus *spp.	*Candida *spp. infection	Noncolonized, uninfected patients	*P*
Patients (*n*)	36	105	720	
Arterial catheter	26.0 ± 19.6	25.1 ± 21.4	14.7 ± 11.7	<0.001
Venous catheter	34.0 ± 38.1	32.6 ± 22.6	17.5 ± 12.4	<0.001
Urinary catheter	35.0 ± 37.5	32.7 ± 2.7	17.8 ± 14.1	<0.001
Mechanical ventilation	26.2 ± 17.2	27.0 ± 20.0	14.6 ± 13.9	<0.001
Total parenteral nutrition	20.7 ± 13.8	21.0 ± 19.0	11.4 ± 13.9	<0.001
Haemodialysis	11.7 ± 11.1	15.7 ± 16.4	15.5 ± 13.1	NS
Neutropenia	8.6 ± 8.2	4 ± 6.1	10.8 ± 9.8	NS
Steroids	29.7 ± 45.2	16.7 ± 14.6	14.1 ± 14.3	0.003
Antibiotic treatment	10.8 ± 5.1	11.5 ± 5.5	9.2 ± 4.7	<0.001

Values are expressed as mean ± standard deviation, unless otherwise stated. ICU, intensive care unit; NS, not significant.

**Table 4:** Characteristics of treatment and outcome in 20 patients with *Aspergillus *spp. infection

Case	APACHE II score at start of treatment	Antifungal agent	Total doses (mg)	Days of treatment	Microbiological eradication	Clinical cure	Outcome
1	18	Liposomal amphotericin B	2450	21	Yes	Yes	Alive
2	29	Itraconazole	-	5	?	No	Death in the ICU
3	17	Liposomal amphotericin B	3100	21	?	Yes	Alive
4	17	Liposomal amphotericin B	2650	17	Yes	Yes	Death in the hospital
5	20	Liposomal amphotericin B	600	4	?	No	Death in the ICU
6	16	Amphotericin B deoxycholate	450	10	No	No	Alive
7	?	Amphotericin B deoxycholate	1050	21	Yes	Yes	Alive
8	23	Amphotericin B deoxycholate	180	5	?	No	Death in the ICU
9	20	Amphotericin B deoxycholate	1050	7	No	No	Death in the ICU
10	19	Amphotericin B lipid complex	2300	9	No	No	Death in the ICU
11	22	Liposomal amphotericin B	3180	21	?	No	Death in the ICU
12	22	Liposomal amphotericin B	3300	15	Yes	Yes	Death in the ICU
13	11	Amphotericin B deoxycholate/liposomal amphotericin B	1200/1200	12/4	No	No	Death in the ICU
14	32	Liposomal amphotericin B	4000	16	Yes	Yes	Death in the ICU
15	45	Amphotericin B lipid complex	2400	8	?	Yes	Alive
16	16	Amphotericin B deoxycholate/amphotericin B lipid complex	350/300	6/2	No	No	Death in the ICU
17	25	Amphotericin B lipid complex/liposomal amphotericin B	3600/6300	14/21	No/Yes	No/Yes	Death in the ICU
18	?	Amphotericin B deoxycholate	420	7	?	No	Death in the ICU
19	34	Amphotericin B deoxycholate	450	7	?	No	Death in the ICU
20	23	Liposomal amphotericin B	2200	11	Yes	Yes	Alive

APACHE, Acute Physiology and Chronic Health Evaluation; ICU, intensive care unit.
